# Toxicity of JQ1 in neuronal derivatives of human umbilical cord mesenchymal stem cells

**DOI:** 10.18632/oncotarget.26127

**Published:** 2018-09-18

**Authors:** Shreeya Bakshi, Christina McKee, Keegan Walker, Christina Brown, G. Rasul Chaudhry

**Affiliations:** ^1^ Department of Biological Sciences, Oakland University, Rochester, MI 48309, USA; ^2^ OU-WB Institute for Stem Cell and Regenerative Medicine, Oakland University, Rochester, MI 48309, USA

**Keywords:** JQ1, BET inhibitor, mesenchymal stem cells, apoptosis, neuronal differentiation

## Abstract

Bromodomain and extra-terminal domain (BET) proteins regulate the transcription of many genes including *c-MYC*, a proto-oncogene, which is upregulated in many types of cancers. The thienodiazepine class of BET inhibitors, such as JQ1, inhibits growth of cancer cells and triggers apoptosis. However, the effects of BET inhibitors on normal cells and mesenchymal stem cells (MSCs), which are important in routine maintenance or regeneration of damaged cells and tissues, are poorly investigated. Previously, we have shown that JQ1 causes human umbilical cord MSCs to undergo cell cycle arrest and neural differentiation. In this study, we determined that JQ1 is more deleterious to neuronal derivatives (NDs) than adipogenic, chondrogenic or osteogenic derivatives of MSCs. NDs treated with JQ1 showed a significant decrease in cell proliferation, viability, and neuronal markers. JQ1 caused cell death through the intrinsic apoptotic pathway in NDs as determined by activation of Caspase 9 and increased expression of Cytochrome C. A comparative analysis showed differential action of JQ1 on MSCs and NDs. The results showed selective neuronal toxicity of JQ1 in NDs but not in the undifferentiated MSCs. These findings suggest a more careful examination of the selection and use of BET inhibitors as therapeutic agents, as they may cause unwanted damage to non-target cells and tissues.

## INTRODUCTION

Bromodomain and extra-terminal domain (BET) proteins bind to acetylated lysine residues of histones [1], play important roles in cellular homeostasis [2] and regulate gene transcription [3]. The BET subfamily of proteins includes BRD2, BRD3, BRD4 and BRDT, which normally act as epigenetic readers [4, 5]. BET inhibitors competitively bind to the acetyl lysine recognition pocket, displacing BET proteins from the chromatin and causing transcriptional changes, which leads to cell cycle arrest and apoptosis [6, 7]. Consequently, inhibition of BET proteins has been extensively investigated as therapeutic agents of certain cancers, inflammatory diseases and metabolic dysfunctions [8–10]. As a result, several BET inhibitors including I-BET762, OTX015, TEN-010, and CPI-0610 have been approved for clinical trials to target cancerous cells [11]. A less stable but more commonly studied BET inhibitor, JQ1 ((S)-tert-butyl2-(4-(4-chlorophenyl)-2,3,9-trimethyl-6H-thieno[3,2-f][1,2,4]triazolo[4,3a][1,4] diazepin-6-yl)acetate), is particularly effective against BRD4 and has been shown to downregulate c-MYC, an oncoprotein involved in cell proliferation and cancer pathogenesis [6]. JQ1 inhibition of BET proteins is known to cause decreased proliferation, cell cycle arrest, and induction of apoptosis in several cancer cell types including pancreatic cancer, leukemia, lymphoma, and triple negative breast cancer [7, 8, 12, 13]. Furthermore, JQ1 has been investigated for treating diseases of the central nervous system since it is capable of crossing the blood brain barrier [14]. JQ1 has been shown to reduce proliferation and induce apoptosis in cells from medulloblastomas and glioblastomas [15, 16]. In addition, it has been tested to treat damaged retinal ganglion cells in a mouse model [17]. JQ1 can also be effective against other neurodegenerative diseases such as Alzheimer’s disease by reducing splenomegaly and neuroinflammation [18].

While anti-cancer and anti-inflammatory properties led to the approval of several inhibitors of BRD4 for drug therapy, their toxicological properties have been poorly investigated. A recent study showed that JQ1 caused memory and other neurological problems in mice, suggesting that BRD4 is important for neurological functions [19]. Another report stated that pharmacological concentrations of JQ1 in mice caused significant weight loss as well as lymphoid and hematopoietic toxicity [20]. In mouse embryonic stem cells, JQ1 induced spontaneous differentiation by downregulating pluripotent genes [21]. We also have previously shown that treatment of JQ1 in mesenchymal stem cells (MSCs) derived from human umbilical cord inhibited growth, caused cell cycle arrest, and interfered with signaling pathways by downregulating expression of WNT [22]. In a clinical study, OTX015, a BET inhibitor that inhibits BRD2, BRD3, and BRD4, was reportedly found to cause thrombocytopenia, anemia, neutropenia, diarrhea, fatigue, and nausea in patients being treated for lymphoma and multiple myeloma [23]. While these limited studies show the effects of JQ1 in pluripotent and multipotent stem cells, the exact mechanism of toxicity is unknown. In this study, we show that JQ1 caused selective toxicity of neuronal derivatives (NDs) of MSCs. These findings have implications in the therapeutic application of BET inhibitors requiring more careful evaluation of their toxicological properties.

## RESULTS

### Examination of JQ1 treatment on cell morphology and growth

**Figure 1:**
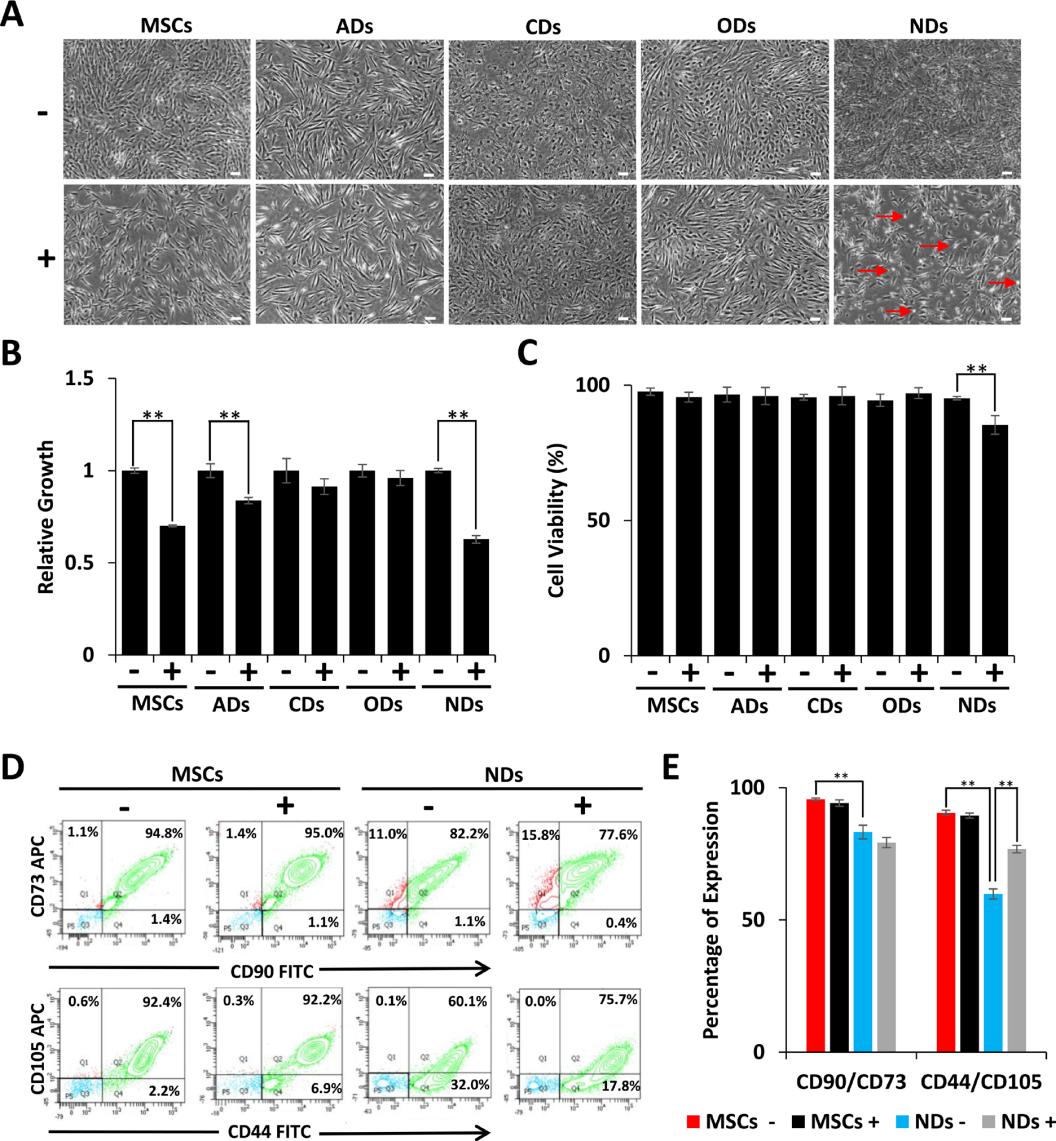
Effect of JQ1 on morphology, viability, and growth of MSCs and derivatives MSCs were cultured in culture medium (CM) or differentiation media for induction into adipogenic, chondrogenic, osteogenic, and neuronal derivatives (ADs, CDs, ODs, and NDs, respectively). (**A**) Cell morphology was visualized by phase contrast microscopy. Scale bars represent 100 µm (Magnification: 4X). Arrows in JQ1 treated NDs point to rounded cells. (**B**) Relative growth of MSCs and their derivatives in the absence or presence of JQ1. (**C**) Graphical representation of the percentage of viable cells as determined by trypan blue staining. (**D**) Representative flow cytometric analysis of MSCs and NDs to determine the MSC specific markers, CD90, CD73, CD44, and CD105. (**E**) Graphical representation of flow cytometric data showing percentage of cells positive for MSC markers. Experiments were performed in triplicate and error bars represent SEM of three independent experiments (*n* = 3). ^*^p < 0.05 and ^**^p <0.01.

Our previous studies have shown that JQ1 inhibited proliferation of MSCs by causing cell cycle arrest and induction of differentiation [22]. In this study, we investigated the effect of JQ1 on derivatives of MSCs. The results depicted in Figure 1A show that MSCs grown in culture medium (CM) displayed typical fibroblastoid morphology but when treated with JQ1 they had flatter morphology and were larger in size as compared to untreated MSCs. MSCs induced to adipogenic, chondrogenic, and osteogenic derivatives (ADs, CDs, and ODs, respectively) did not show significant morphological changes upon treatment with JQ1 (Figure 1A). MSCs cultured in neural induction medium (NM) differentiated into NDs, exhibited cellular extensions, and had large nuclei, typical of early neural progenitors [24, 25]. However, these neural extensions were lost upon treatment with JQ1. In addition, JQ1 caused cell rounding and loss of adhesion, while the remaining adherent cells exhibited fibroblastoid morphology (Figure 1A). Although JQ1 adversely affected cell proliferation of MSCs, ADs, and NDs (Figure 1B), only NDs showed a significant reduction in cell viability (10%) (Figure 1C). On the other hand, an insignificant decrease in the viability of undifferentiated MSCs in the presence of JQ1 was observed. JQ1 also did not significantly affect the viability of ADs, CDs and ODs (Figure 1C), indicating that MSCs differentiated into the neural lineage were selectively sensitive to JQ1.

Because the majority of the NDs treated with JQ1 remained viable, we wondered if not all of the MSCs differentiated into NDs. We investigated the expression of MSC surface markers by flow cytometry, and the results depicted in Figure 1D and 1E showed a reduction in CD90/CD73 expression from 95% to 82% in MSCs and NDs, respectively. Furthermore, CD44/CD105 expression decreased from 92% to 60% when MSCs were induced to neural differentiation. This significant loss of MSC markers in NDs suggested that only a specific population of MSCs underwent differentiation.

The expression of MSC markers remained almost the same in undifferentiated MSCs cultured in the absence or presence of JQ1. However, when MSCs were induced to differentiate into NDs and treated with JQ1, CD90/CD73 expression was decreased insignificantly from 82% to 77% but CD44/CD105 expression increased from 60% to 75%. Thus, suggesting that JQ1 was selectively deleterious to differentiated cells.

### Effect of JQ1 on the expression of neural markers

**Figure 2:**
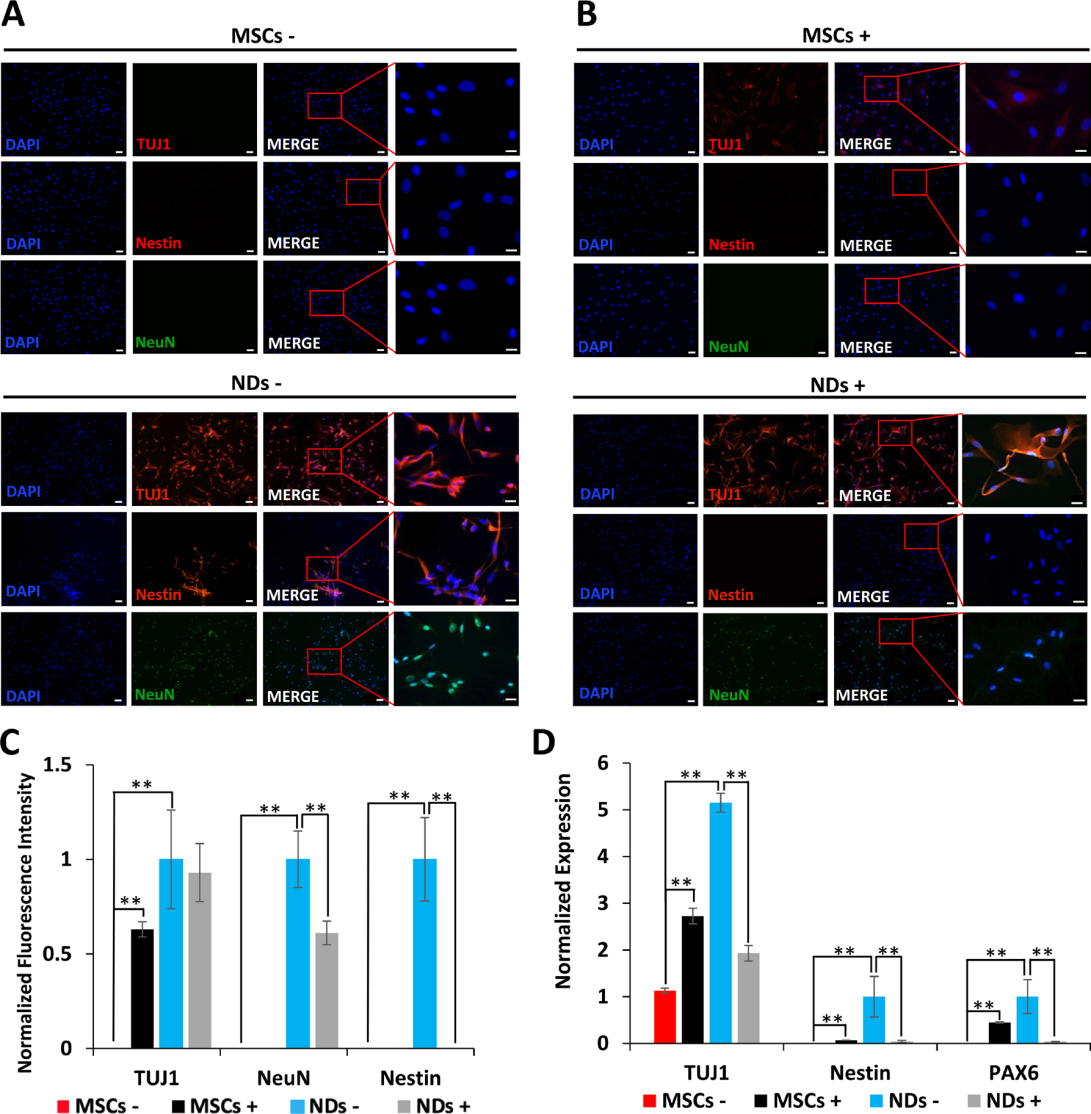
Effect of JQ1 on expression of neural markers MSCs and NDs were untreated (−) or treated (+) with JQ1 for 48 hours. (**A** and **B**) Immunocytochemical analysis of expression of neural proteins TUJ1, Nestin, and NeuN, in MSCs and NDs in the absence or presence of JQ1, respectively. Scale bars represent 50 μm (Magnification: 10X) and 20 μm in high magnification merged inserts (Magnification: 40X), respectively. (**C**) Quantification of normalized fluorescent intensities of neural proteins in MSCs and NDs treated with and without JQ1 using ImageJ software. (**D**) Transcriptional analysis of neural genes, *TUJ1*, *Nestin*, and *PAX6* as determined by qRT-PCR. Experiments were performed in triplicate and error bars represent SEM of three independent experiments (*n* = 3). ^*^p < 0.05 and ^**^p < 0.01.

The results depicted in Figure 2A show expression of early neurogenic proteins, TUJ1, Nestin, and NeuN, in NDs but not MSCs further confirming that MSCs were induced to the neuronal lineage in NM. Consistent with our previous findings [22], treatment of JQ1 resulted in an increase in TUJ1 expression in MSCs. However, JQ1 caused a significant decrease in the expression of Nestin and NeuN, but not TUJ1 in NDs (Figure 2B and 2C). We then investigated the transcriptional expression of neural markers, *TUJ1*, *Nestin,* and *PAX6* using quantitative reverse transcriptase polymerase chain reaction (qRT-PCR). The results described in Figure 2D show loss of expression of neural genes in NDs upon treatment with JQ1, suggesting the selective toxicity of differentiated neuronal cells but not the undifferentiated cells (MSCs).

### Analysis of cell death

**Figure 3:**
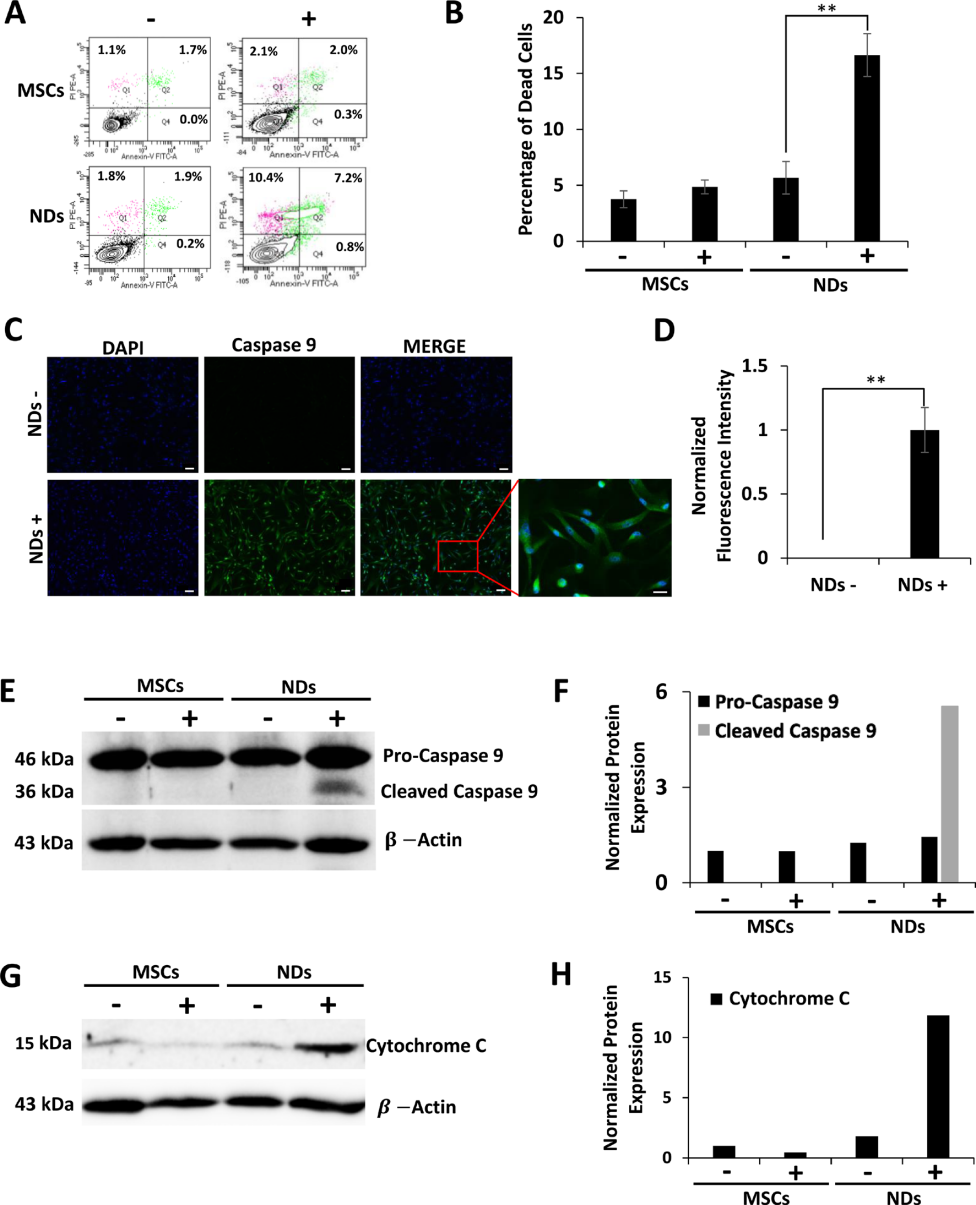
Effect of JQ1 on the expression of Caspase 9 and Cytochrome C MSCs and NDs untreated (−) and treated (+) with JQ1 for 48 hours and subjected to analysis. (**A**) Representative flow cytomeric plots of cells stained with Annexin-V/FITC and PI. (**B**) Graphical representation of the average percentage of dead cells as determined by flow cytometry, error bars represent SEM of three independent experiments (*n* = 3). (**C**) Immunocytochemical analysis of Caspase 9 showing protein expression in NDs treated with JQ1. Scale bars represent 50 μm (Magnification: 10X) and 20 μm in high magnification merged insert (Magnification: 40X), respectively. (**D**) Quantification of normalized fluorescent intensity of Caspase 9 expression in NDs using ImageJ software. ^*^p < 0.05 and ^**^p < 0.01. (**E**) Western blotting analysis of Caspase 9 protein expression showing cleaved Caspase 9 at 36 kDa in the JQ1 treated NDs. **(F**) Quantification of Caspase 9 protein expression normalized to β-Actin using ImageJ software. (**G**) Western blotting analysis showing Cytochrome C protein expression. (**H**) Quantification of Cytochrome C protein expression normalized to β-Actin using ImageJ software.

The loss of cell viability in NDs exposed to JQ1 was also evaluated using an apoptosis assay. The results shown in Figure 3A and 3B depict representative flow cytometric analysis of Annexin-V and propidium iodide (PI) staining and the average percentage of dead cells, respectively. A significantly higher percentage of dead cells was observed in JQ1 treated NDs (16.7%) as compared to untreated NDs (Figure 3B). The dead cells stained with both Annexin-V and PI were likely to be in the late stages of apoptosis. Based on the fact that the adherent cells had fibroblastoid morphology after JQ1 treatment and expressed MSC markers as shown above, the loss of viability of NDs was confirmed via apoptosis rather than random cell death.

To further understand the apoptosis induced in NDs by JQ1, we investigated the expression of proteins involved in cell death. The results of the immunocytochemical analysis given in Figure 3C and quantified in Figure 3D showed that NDs treated with JQ1 had increased fluorescence expression of Caspase 9 as compared to the untreated control. Higher expression of Caspase 9 was confirmed by western blot analysis (Figure 3E and 3F). Furthermore, JQ1 treated NDs showed activation of Caspase 9 as evident by the presence of the 36 kDa cleaved protein. In addition, western blot results shown in Figure 3G and 3H indicated an increase in the expression of Cytochrome C in NDs treated with JQ1 as compared to the untreated control cells.

**Figure 4:**
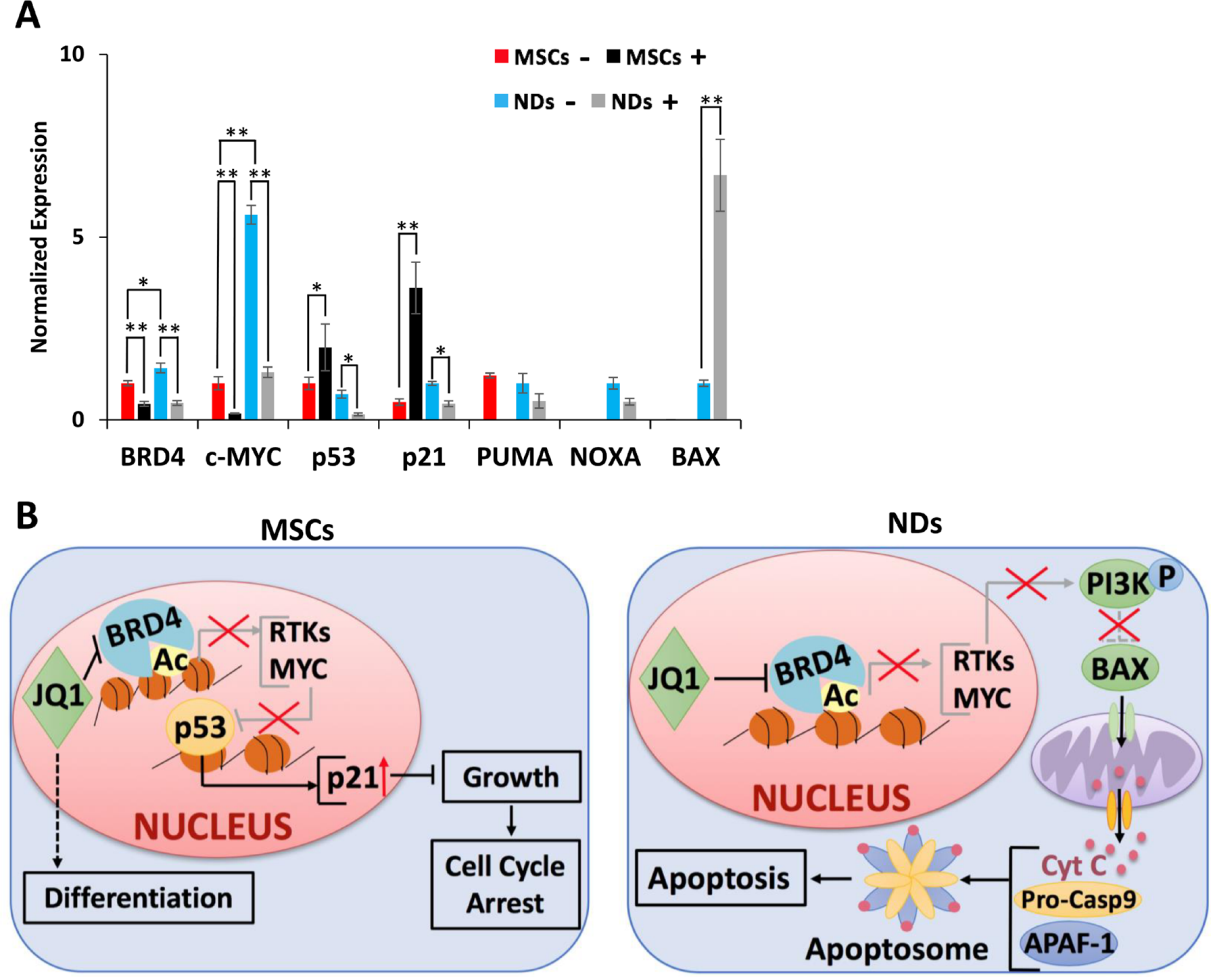
Proposed mechanism of action of JQ1 **(A**) Transcriptional analysis of *BRD4*, *c*-*MYC*, *p53*, *p21*, *PUMA*, *NOXA*, and *BAX* in MSCs and NDs untreated (−) and treated (+) with JQ1 as determined by qRT-PCR. Experiments were performed in triplicate and error bars represent SEM of three independent experiments (*n* = 3). ^*^p < 0.05 and ^**^p < 0.01. **(B**) JQ1 caused p21 mediated cell cycle arrest and differentiation in MSCs, and resulted in Caspase 9 mediated intrinsic apoptosis in NDs.

Further investigation of the action of JQ1 showed upregulation of *BRD4* and *c-MYC* genes in NDs in comparison to MSCs, but downregulation of these genes upon JQ1 treatment (Figure 4A). In MSCs, JQ1 caused upregulation of *p53* and *p21*, suggesting cell cycle arrest as reported previously [22]. In NDs, JQ1 caused a significant downregulation of *p53* and *p21* but upregulation of *BAX*, while there was no significant difference in expression of *PUMA* and *NOXA* (Figure 4A). Based on these results, we proposed a mechanism of action of JQ1 in MSCs and NDs as depicted in Figure 4B. JQ1 caused p21 mediated cell cycle arrest and induced differentiation in MSCs but resulted in intrinsic apoptosis in NDs. Taken together, these results suggest that JQ1 differentially affected MSCs and NDs.

## DISCUSSION

Several BET inhibitors have been approved as anti-cancer agents for leukemia, lymphoma, and multiple myeloma [23, 26]. They have been shown to downregulate *c-MYC* and decrease cell proliferation [6, 27, 28]. BET inhibitors can also cause apoptosis of cancer cells by downregulating receptor tyrosine kinase (RTK) signaling pathways [29]. We have previously shown that JQ1 caused not only cell cycle arrest but differentiation of human umbilical cord MSCs [22].

Studies have shown that JQ1 suppresses differentiation in adipogenic, chondrogenic, osteogenic, and myogenic cells [30–34]. To examine the effect of JQ1 on neural differentiation, MSCs were cultured in neural induction medium, NM. The results indicated that NM induced MSCs to differentiate into NDs, since they expressed PAX6 and Nestin, characteristic of neural stem cells [35, 36], as well as NeuN and TUJ1, markers of neuronal precursors and neurons [37, 38]. A previous report showed that BET inhibition by JQ1 enhanced differentiation of mouse neural progenitor cells into neurons but suppressed cell cycle progression and gliogenesis [39]. In contrast, our results showed that JQ1 caused the loss of cellular extensions in NDs, followed by rounding of cells. These results are similar to previously reported morphological changes observed with other JQ1 sensitive cells, including pancreatic stellate cells and human foreskin fibroblasts [40, 41].

Our preliminary investigation of MSC derivatives showed that JQ1 did not have a significant effect on the morphology of ADs, CDs, and ODs. While JQ1 adversely affected the proliferation of both MSCs, ADs and NDs, only NDs showed a significant decrease in cell viability following exposure to JQ1. This is consistent with our previous study whereby JQ1 inhibited proliferation by cell cycle arrest but did not cause cell death in MSCs [22].

Induction of neural differentiation of MSCs yielded early neuronal derivatives, while a majority of the cells remained undifferentiated as they expressed MSC surface markers. In comparison to MSCs, NDs exhibited only a 15.5% and 31% decrease in CD90 and CD105 expression, respectively. Interestingly, the percentage of cells expressing CD105 was higher in NDs following JQ1 treatment. Evidently, JQ1 was selectively toxic to neuronal cells but not to undifferentiated cells, which may explain the increase in the expression of MSC markers and decrease in neural markers observed in NDs treated with JQ1.

Furthermore, the induction of MSCs into NDs resulted in the increased expression of neuronal markers, *TUJ1*, *Nestin*, and *PAX6*, as well as *BRD4* and *c-MYC*. BRD4 has been shown to activate transcription in neurons and is typically expressed in cells positive for NeuN but not GFAP [19]. Additionally, c-MYC has been shown to promote neuronal differentiation [42]. JQ1 treatment led to downregulation or loss of these markers. Again, this was presumably due to the selective toxicity of JQ1 to NDs.

We further investigated JQ1 induced cell death in NDs. The results indicated increased expression of Caspase 9 in the JQ1 treated but not the untreated NDs. In addition, the western blot analysis showed activation of Caspase 9 but not Caspase 8 (data not shown), indicating that death of neuronal cells was caused by Caspase 9 mediated apoptosis. This is in line with previous reports that showed that JQ1 activated Caspase 9 in established cancer cell lines, including hepatocellular carcinoma and glioblastoma cells [43, 44]. In addition, the results indicated upregulation of Cytochrome C, which is released by the mitochondria to cause cleavage of Caspase 9 in JQ1 treated NDs.

Further investigation of the mechanism of action of JQ1 in NDs showed downregulation of *p53* and *p21*. This corresponded with the near significant downregulation of *NOXA*, and *PUMA*, which are transcriptional targets of p53 [45–47]. We also investigated the expression of *BAX,* a pro-apoptotic protein that triggers intrinsic apoptosis through the release of Cytochrome C [48, 49], and found it to be significantly upregulated in NDs treated with JQ1. These results indicated that NDs are sensitive to JQ1, and undergo Caspase 9 mediated apoptosis. JQ1 sensitivity may be due to high levels of *c-MYC* expression in NDs following induction of neuronal differentiation. In fact, JQ1 is well known to cause downregulation of c-MYC [6, 28, 50] and has been shown to significantly decrease cell proliferation and preferentially induce apoptosis in medulloblastoma derived cell lines expressing high levels of MYC [16].

In sensitive cells, JQ1 treatment inhibits the BRD4 mediated transcription of RTKs [29, 51], resulting in the inactivation of the phosphoinositide 3-kinase (PI3K)/Akt pathway, which leads to the downstream dephosphorylation of BAX, thus allowing it to enter the mitochondria resulting in the release of Cytochrome C [52]. This is consistent with the Caspase 9 mediated cell death observed in NDs. On the other hand, transcriptional analysis showed upregulation of *p53* and *p21* in MSCs treated with JQ1, which is consistent with our previous study indicating that MSCs are undergoing cell cycle arrest [22]. We propose that JQ1 treated MSCs are undergoing p21 mediated cell cycle arrest, which is in agreement with other studies showing that JQ1 induced G1 cell cycle arrest caused by upregulation of p21 [15, 53–55].

Overall, these results led us to propose the mechanism of action of JQ1 in MSCs and NDs as depicted in Figure 4B. Briefly, JQ1 caused cell cycle arrest and differentiation in MSCs, but neural toxicity in NDs. These results are likely to prompt further investigation into the molecular mechanism of action of JQ1. Furthermore, they suggest a more careful evaluation of the use of BET inhibitors as therapeutic agents, since they may cause unwanted damage to non-target cells and tissues.

## MATERIALS AND METHODS

### Cell culture

Human umbilical cord MSCs were isolated and characterized as previously described [56]. MSCs (Passage 7) were grown in culture medium (CM) containing high glucose Dulbecco’s modified Eagle’s medium (DMEM) with 4500 mg/L glucose and 2 mM L-glutamine (Fisher Scientific, Pittsburgh, PA, USA). CM was supplemented with 10% fetal bovine serum (FBS) (VWR International, Radnor, PA, USA) and 5.6% of antibiotic solu tion (0.1% gentamicin, 0.2% streptomycin, and 0.12% penicillin) (Sigma Aldrich, St. Louis, MO, USA). MSCs were differentiated into ADs using high glucose DMEM containing 0.5 μM IBMX (Sigma), 1 μM dexamethasone (Sigma), 10 μM insulin (PeproTech, Rocky Hill, NJ, USA), and 200 μM indomethacin (Sigma). MSCs were differentiated into CDs using high glucose DMEM supplemented with 20 ng TGFβ1 (PeproTech), 10 ng insulin (PeproTech), 100 nM dexamethasone (Sigma), and 100 μM ascorbic acid (Sigma). Differentiation into ODs was achieved using high glucose DMEM supplemented with 0.1 μM dexamethasone (Sigma), 10 μM β-glycerophosphate (Sigma), and 50 μM ascorbate-phosphate (Sigma). To differentiate MSCs into NDs, MSCs were cultured in neural induction medium (NM) containing neurobasal medium (Fisher Scientific), supplemented with 2 mM L-glutamine, 2% B27 (Fisher Scientific), 10^−5^ M retinoic acid (Sigma), 100 μM ascorbic acid (Sigma), and 5 µM IBMX (Sigma), 20 ng/mL NGF, 30 ng/mL EGF, and 10 ng/mL bFGF (PeproTech). Cells were grown in a 5% CO_2_ incubator.

### Treatment of cells with JQ1

MSCs were cultured in CM as controls. For differentiation, MSCs were grown in culture plates (2.5 × 10^4^ cells/25 flasks) in cell specific media for 2 (NDs) or 5 (ADs, CDs, ODs, and NDs) days. They were then treated in the absence or presence of 500 nM JQ1 (Bradner’s Laboratory, Harvard Medical School, Boston, MA, USA) dissolved in dimethyl sulfoxide (Fisher Scientific) and further incubated for 48 hours before analysis for morphological and biological changes.

### Determination of cell viability and proliferation

After MSCs and their derivatives were cultured in their respective media for 2 (MSCs and NDs) or 5 (ADs, CDs, and ODs) days, they were then treated in the absence or presence of JQ1 for 48 hours followed by staining with trypan blue solution (Sigma) to determine cell proliferation and viability. Relative growth was normalized to untreated controls. The stained cells were considered non-viable and counted using a hemocytometer.

### MSC characterization using flow cytometry

MSCs were cultured in CM or NM for 2 days, treated in the absence or presence of JQ1 for 48 hours, dissociated with TrypLE (Life Technologies, Carlsbad, CA, USA), centrifuged, and re-suspended in PBS. Cells were incubated with mouse antibodies against specific markers including CD90 and CD44 (conjugated with FITC), and CD73 and CD105 (conjugated with APC) (Fisher Scientific) and analyzed by flow cytometry and Diva Software (BD Canto II, BD Biosciences, San Jose, CA, USA).

### Apoptosis assay

MSCs were cultured in CM and NM for 2 days, treated in the absence or presence of JQ1 for 48 hours, and then subjected to apoptosis assay. In brief, cells suspended in 100 μL of Annexin-V binding buffer were stained with 5 μL of Annexin-V/FITC (Biolegend, San Diego, CA, USA) and 10 μL of propidium iodide (PI, Sigma, St. Louis, MO, USA). The stained samples were analyzed by flow cytometry. Cells that were positive for Annexin-V staining only were considered apoptotic while cells that were positive for PI were considered necrotic, and cells positive for both Annexin-V and PI were defined as late apoptotic. Viable cells were negative for both Annexin-V and PI staining.

### Immunocytochemistry analysis

MSCs cultured in CM and NM for 5 days were then treated in the absence or presence of JQ1 for 48 hours before analysis. For immunocytochemistry, cells were fixed with 4% paraformaldehyde for 10 minutes, permeabilized with 0.5% TritonX100 (Sigma) for 10 minutes, and blocked with 2% bovine serum albumin (Sigma) dissolved in PBS for 1 hour to block non-specific binding. Cells were then incubated with primary antibodies including TUJ1 (Santa Cruz Biotechnology, Dallas, TX, USA), NeuN (Invitrogen, Carlsbad, CA, USA), Nestin (Santa Cruz Biotechnology), or Caspase 9 (Santa Cruz) overnight at 4° C. Primary antibodies were then removed, cells were washed with PBS three times and stained with the respective secondary antibodies (Cy3-labelled goat anti-mouse IgG (KPL, Gaithersburg, MD, USA), and Alexa Fluor 488 goat anti-rabbit IgG (Fisher Scientific) for 1 hour at room temperature. Secondary antibodies were then removed and cells were washed with PBS and stained with 4′,6-diamidino-2-phenylindole (DAPI, Fisher Scientific) to stain the nuclei. Stained cells were analyzed using fluorescence microscopy (NIKON Instruments Inc., Melville, NY, USA). Protein expression was quantified with ImageJ software for the corrected total cell fluorescence (CTCF) using the following equation: CTCF = Integrated Density – (Area of selected cell x Mean fluorescence of background readings). Fluorescence intensities were normalized to the expression of NDs without JQ1.

### qRT-PCR

MSCs cultured in CM and NM for 5 days were treated in the absence or presence of JQ1 for 48 hours. Total mRNA was isolated from these cells using the GeneJET RNA purification kit (Fisher Scientific) by following the instructions given by manufacturer. RNA was purified by incubating the isolated RNA at 37° C for 30 minutes with DNase (Promega, Madison, WI, USA) in a thermocycler (MJ Research PTC-100 Thermal Cycler; GMI, Ramsey, MN, USA). The purified RNA was used to synthesize cDNA by using BioRad iScript kit (BioRad, Hercules, CA, USA). Sso-Advanced Universal SYBR Green Supermix Kit (BioRad) was used for qRT-PCR using the CFX96 Real-Time System (BioRad) with 5 μL of SYBR green, 3 μL of distilled water, 1 μL of cDNA that is diluted 1:10 in distilled water, and 0.5 μL of forward primer and reverse primer. *GAPDH* and *β*-*ACTIN* were used as reference genes to normalize the amplification of the target genes. Primer sequences are listed in Table 1.

**Table 1:** List of human primer sequences used in qRT-PCR

Gene	Primer Sequence		
	Forward (5′-3′)	Reverse (5′-3′)	Product Length
*TUJ1*	GGCCAAGTTCTGGGAAGTCA	CGAGTCGCCCACGTAGTTG	70
*Nestin*	GAAACAGCCATAGAGGGCAAA	TGGTTTTTCCAGAGTCTTCAGTGA	168
*PAX6*	CTTTGCTTGGGAAATCCGAG	AGCCAGGTTGCGAAGAACTC	103
*BRD4*	AGGCAAAAGGAAGAGGACG	CGATGCTTGAGTTGTGTTTGG	86
*c-MYC*	CGGAACTCTTGTGCGTAAGG	CTCAGCCAAGGTTGTGAGGT	123
*p53*	GGGAGCACTAAGCGAGCA	ACGCCCACGGATCTGAAG	102
*p21*	GCAGACCAGCATGACAGATTT	GGATTAGGGCTTCCTCTTGGA	70
*PUMA*	GACGACCTCAACGCACAGTA	CTAATTGGGCTCCATCTCG	147
*NOXA*	GAGATGCCTGGGAAGAAGG	TTCTGCCGGAAGTTCAGTTT	125
*BAX*	GGGTGGTTGGGTGAGACTC	AGACACGTAAGGAAAACGCATTA	191
*GAPDH*	GAAGGTGAAGGTCGGAGTC	GAAGATGGTGATGGGATTTC	226
*β-ACTIN*	AATCTGCGACCACACCTTCTAC	ATAGCACAGCCTGGATAGCAAC	170

### Western blot analysis

MSCs cultured in CM or NM for 5 days and then treated with or without JQ1 were lysed for protein using RIPA buffer (Sigma) and quantified using the Pierce 660 nm protein assay on the NanoDrop 1000 spectrophotometer (Fisher Scientific). The lysate (30 μg of total proteins) was resolved using SDS-PAGE with 12% resolving gel and 6% stacking gel and transferred to a nitrocellulose membrane (BioRad) at a continuous current of 100V for 90 minutes. For antibody staining, the membrane was blocked with 5% nonfat dry milk dissolved in TBS 1X containing 0.1% Tween-20 (TBST) for 30 minutes and incubated with primary antibodies (Caspase 9, Cytochrome C, β-Actin; Santa Cruz Biotechnology) at a 1:500 dilution in the blocking solution overnight at 4° C. The membrane was then washed with TBST and incubated with the secondary antibody conjugated with HRP (Santa Cruz Biotechnology) at a 1:10,000 dilution in the blocking solution for 2 hours at room temperature. After washing with TBST, the blot was stained with BioRad chemiluminescence for 5 minutes and bands were visualized using a chemidoc (BioRad). Band intensities were quantified using ImageJ software (NIH, Bethesda, MD, USA) and normalized to β-Actin.

### Statistical analysis

Data are presented as the mean ± standard error of the mean (SEM). All experiments were performed in triplicate and three independent experiments were carried out. One-way ANOVA analysis was performed and results with a *p*-value ≤ 0.05 were considered statistically significant (^*^p < 0.05 and ^**^p < 0.01) as compared to untreated control cells. All analyses were performed using SPSS version 11.5 (SPSS Inc.).

## Abbreviations

BET: Bromodomain and extra-terminal domain
JQ1: (S)-tert-butyl2-(4-(4-chlorophenyl)-2,3,9-trimethyl-6H-thieno[3,2 f][1,2,4]triazolo[4,3a][1,4] diazepin-6-yl)acetate
MSCs: Mesenchymal stem cells
CM: Culture medium
ADs: Adipogenic derivatives
CDs: Chondrogenic derivatives
ODs: Osteogenic derivatives
NM: Neural induction medium
NDs: Neuronal derivatives
qRT-PCR: Quantitative reverse transcriptase polymerase chain reaction
PI: Propidium iodide
RTK: Receptor tyrosine kinase
PI3K: Phosphoinositide 3-kinase
FBS: Fetal bovine serum
SEM: Standard error of the mean
